# Returning home: The role of expectations in re‐entry adaptation

**DOI:** 10.1111/aphw.12361

**Published:** 2022-04-05

**Authors:** Nicolas Geeraert, Colleen Ward, Paul H. P. Hanel

**Affiliations:** ^1^ University of Essex Colchester UK; ^2^ Centre for Applied Cross‐Cultural Research Victoria University at Wellington Wellington New Zealand

**Keywords:** acculturation, adaptation, expectation, re‐entry, return, sojourn

## Abstract

Returning home after a study abroad experience can be challenging. In the current research, we examine the discrepancy between adaptation expectations and experience in a longitudinal sojourner study (*N* = 1319; *M*_age_ = 17 years; 70% female). Returnees adaptation expectations were assessed prior to returning home, followed by post return measures of adaptation experiences and general well‐being. Overall, returnees reported higher levels of re‐entry adaptation than anticipated. According to the accuracy hypothesis, unmet expectations will be associated with lower well‐being. In contrast, the directional hypothesis suggests that unmet expectations will negatively impact on well‐being, but only if the expectation is undermet. Well‐being on return was regressed on pre‐travel adaptation expectations and adaptation experience on re‐entry. Polynomial regression and Response Surface Analyses were conducted for two outcome variables (stress and satisfaction with life), two types of adaptation (psychological and sociocultural), and at different time points (approximately 2 weeks and 6 months after return). Results consistently show that larger discrepancies were associated with lower well‐being for negative mismatches (when expectations were undermet). For positive mismatches, if adaptation was better than expected, well‐being was higher. Congruence between expectation and experience were not associated with well‐being. Thus, across analyses, results supported a directional hypothesis.

## INTRODUCTION

*It is common for students returning from living and studying abroad to have a difficult time adjusting … The primary cause of “reverse culture shock” is a lack of realistic expectations* (Office of Global Education, Georgetown University, n.d.).

*It is often more challenging for students to readjust to their home culture upon return from an international or cross‐cultural experience than to adjust to a new culture upon arrival in a foreign community … Many students are challenged to return to a place where they expect to feel comfortable, and instead experience feelings of frustration and misunderstandings. Students often have an idealized view of home and are shocked when reality does not meet their expectations* (Young, 2014, pp. 59–60).

Both global education experts and counseling professionals have long recognized that returning home after study abroad can be an unsettling experience and that expectations play a key role in shaping the re‐entry process (Arthur, 2003; Gaw, 2000; Sussman, 2000; Young, 2014). Just how expectations impact returnees' psychological well‐being, however, remains in question. On one hand, realistic expectations have been advocated as a means of fostering positive social and psychological outcomes for international students returning home. On the other hand, it has been argued that the accuracy of expectations is less important than the direction of the discrepancy between expectations and experiences. Specifically, it has been suggested that a positive mismatch, that is, re‐entry experiences that are better than expected, result in higher levels of well‐being while a negative mismatch is associated with poorer psychological outcomes (Martin & Harrell, 2004).

**FIGURE 1:**
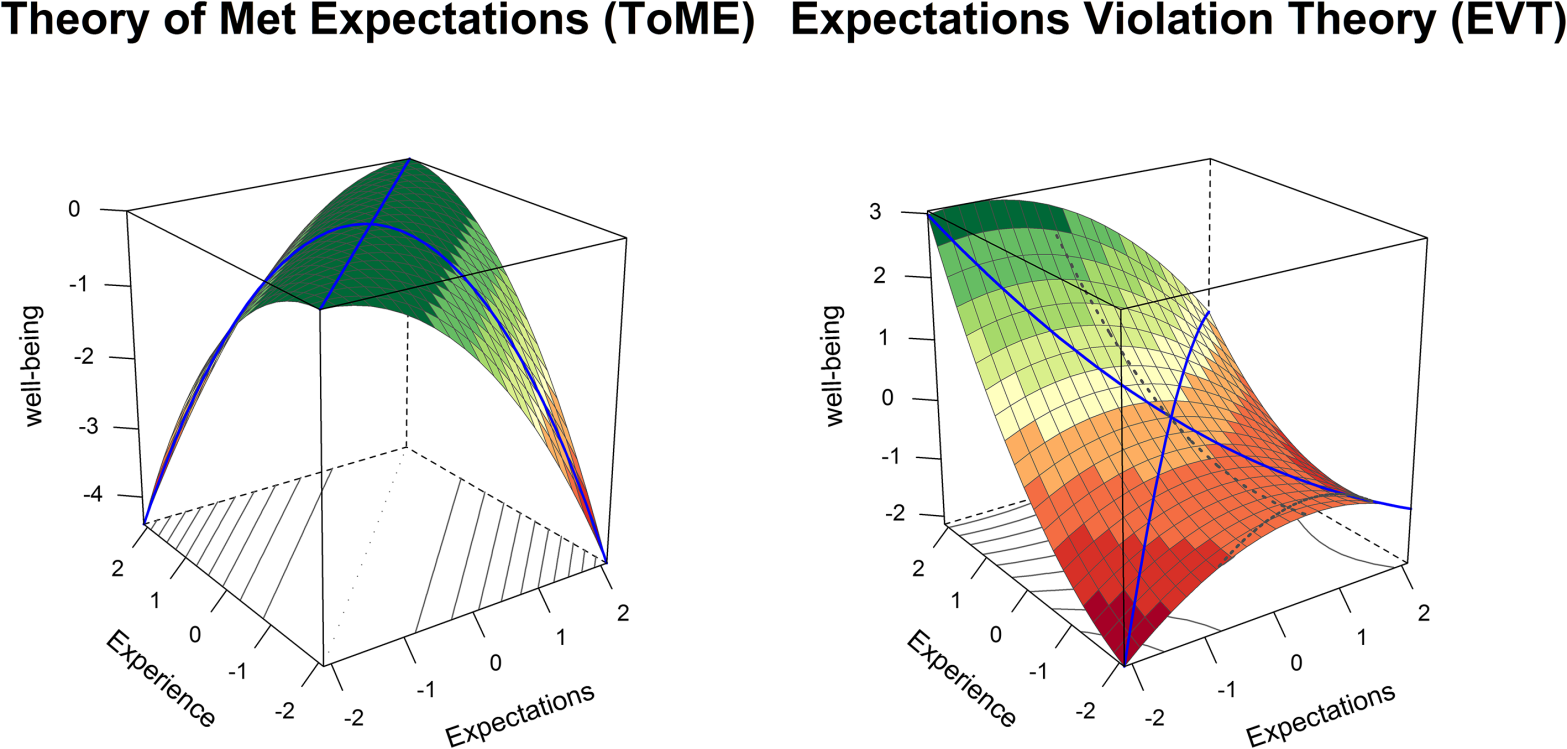
Prediction of the theory of met expectations (left panel) and of the expectations violation theory (right panel)

The conceptual frameworks for understanding and interpreting the relationship between re‐entry expectations and experiences have been primarily drawn from organisational psychology and intercultural communication research (Caligiuri et al., 2001; Martin & Harrell, 2004). The Theory of Met Expectations (ToME), arising from organisational psychology, proposes that the greater congruency between individuals' expectations and experiences, the higher their job satisfaction and adjustment (Caligiuri et al., 2001; Porter & Steers, 1973; Wanous, 1992; Wanous et al., 1992). All three variables, expectation, experience, and outcome, can be plotted in a three‐dimensional space (see Figure 1, left panel). The ToME would predict an outcome, such as well‐being, to be high, when expectations and experience align, as indicated by the green part of the plane. In contrast, well‐being would be low when either expectations or experience were low, but not the other, as indicated by the yellow and red parts of the plane. Black's (1992) study of repatriates supported the ToME partially. Overall returnees reported the highest level of job performance and adjustment when their job expectations were met, rather than undermet or overmet. In terms of general expectations about living conditions, however, overmet expectations were nonetheless associated with better adjustment outcomes.

In contrast to the ToME, the Expectations Violation Theory (EVT), emerging from communication theory and research, asserts that disconfirmed, compared to accurate, expectations can result in more positive outcomes, depending on the nature of the discrepancy. The theory makes a number of related predictions (Burgoon, 1995, 2015): (1) positive violations (when circumstances or experience are better than expected) lead to more favorable outcomes, (2) negative violations (when circumstances or experience are worse than expected) lead to less favorable outcomes, and (3) positive violations lead to more favorable outcomes than negative violations. These predictions were used to visualize the model (see Figure 1, right panel). Thus, an outcome, such as well‐being may be expected to be highest when experience is more positive than expectations. In line with EVT, Rogers and Ward's (1993) study with returning exchange students found that realistic expectations about social difficulties were unrelated to psychological adjustment on return, but when difficulties were greater than expected, larger discrepancies were associated with greater psychological distress.

While qualitative research has also considered the role of expectations in the re‐entry process (e.g. Bochner et al., 1980; Christofi & Thompson, 2007), to the best of our knowledge, the research by Black (1992) and Rogers and Ward (1993) are the only two quantitative studies to explore pre‐return expectations and post‐return experiences in relation to returnees' adjustment and psychological well‐being. Unfortunately, both are constrained by notable limitations. Black (1992) collected expectations data retrospectively after repatriates had returned home, and the Rogers and Ward (1993) study had a very small (*N* = 20) sample. Interestingly, neither theory (ToME or EVT) are explicit on the content of expectations. Thus, unsurprisingly, these studies measured expectations in different domains, either job expectations in the work context (Black, 1992), or expected social difficulties in sojourners (Rogers & Ward, 1993). In the context of sojourning, the distinction between sociocultural adaptation (the ability to fit in) and psychological adaptation (how sojourners *feel*) provides an excellent framework (Searle & Ward, 1990; Ward & Kennedy, 1996) to test the role of expectations on re‐entry.

Studies of expectations and experiences during cross‐cultural transitions are more common for outward‐bound travelers although the corpus of work is still small, and its potential for clarifying the relative benefits of realistic versus overmet expectations is limited as findings are not consistent across studies. Weissman and Furnham's (1987) research with American expatriates in London failed to find support for the accuracy hypothesis; however, Mähönen and Jasinskaja‐Lahti's (2013) study of ethnic immigrants to Finland found conditional support for the benefits of realistic expectations. The most positive well‐being outcomes emerged when immigrants' expectations were both positive and in accordance with their experiences. At the same time, the direction of discrepancies between expectations and experiences was important; psychological well‐being was low when immigrants' anticipated fewer social and cultural difficulties than experienced. Mixed findings were also reported by Negy et al. (2009) in their study of Latino immigrants in the United States. Although there was little difference in the stress levels between those with met and overmet expectations, there was some evidence that undermet expectations were associated with greater stress. Overmet expectations have been shown to be associated with greater satisfaction with overseas experiences (Black & Gregersen, 1991; Martin et al., 1995); however, only Caligiuri et al.'s (2001) study with expatriates clearly demonstrated that cross‐cultural adjustment varies as a function of the degree to which expectations are overmet.

As the empirical approaches to research on expectations and experiences during cross‐cultural transition have been highly varied and the findings mixed, it has been difficult to assess the evidence base for the ToME and the EVT. Moreover, in general, the overall quality of re‐entry research has been relatively poor. Geeraert et al. (2021) identified five major shortcomings in this body of research: (1) few studies have explicitly tested accuracy and directional discrepancies as competing hypotheses, which limits the interpretation of findings; (2) the techniques for classifying the relationship between expectations and experiences and the accompanying statistical analyses used in these studies have often confounded met expectations with positive violations because they have failed to assess the independent influences of both the magnitude (the degree of the discrepancy) and the direction (positive vs. negative mismatch) of expectation–experience discrepancies; (3) most studies have relied upon retrospective reporting of expectations, which is known to be unreliable (Erdfelder et al., 2007); (4) many studies operate with relatively small sample sizes (*N*s < 80) and are underpowered; and (5) all but one study (Caligiuri et al., 2001) have been limited to research participants either from a single country of origin or traveling to a single destination country, even though cultures of origin and destination are known to exert influences on sojourners' adaptation (Geeraert et al., 2019). The present study of international student re‐entry addresses these limitations.

### The present study

The current study builds on previous research contrasting the accuracy and directional hypotheses (from ToME and EVT), using data from a multinational longitudinal study of exchange students (see Demes & Geeraert, 2015; Geeraert et al., 2019). Expectations about re‐entry adaptation were measured about 1 month prior to travel, and their adaptation experience and well‐being were measured approximately 2 weeks and 6 months after return. Sojourners' return expectations and experiences were examined in relation to their behavioral re‐adaption in their home culture (sociocultural adaptation) and their affective state (psychological adaptation) (Searle & Ward, 1990). The accuracy and directional hypotheses from the ToME and the EVT are examined by assessing whether congruence between expectation and experience resulted in higher well‐being (ToME) or whether more positive experiences than expected was linked with higher well‐being (EVT) with well‐being assessed in terms of less stress and greater life satisfaction. Crucially, each of these predictions can be tested using polynomial regression and response surface analysis, as we outline in detail below.

## METHOD

### Design and participants

Data were analyzed from a longitudinal acculturation project (see Demes & Geeraert, 2015; Geeraert et al., 2019),^1^ in which 2480 young adults (age: *M* = 17.0 years, *SD* = 1.4 years; 70% female), participating in an intercultural exchange program, were surveyed over an 18‐month period, from 2 months before to approximately 6 months after the exchange. All participants were registered with AFS Intercultural Programs,^2^ a non‐profit, volunteer‐based organisation offering international exchange programs. Typically, students are placed with a host family for the duration of their 8‐ to 10‐month stay abroad, and during this time, they enroll at a local high school. Each sojourner in this study was traveling from 1 of 46 different home countries to 1 of 51 different host destinations.

Participants and their parents were informed about the study prior to participation, with consent obtained from participants and their parents (for minors). Ethics for the study was granted by the University of Essex' Ethics Committee.

Sojourners were surveyed a total of nine times, but for the purpose of the current research, we focused on data collected during three waves (t6, t7, and t9) because our measures of interest were assessed at only these times. Waves occurred approximately 1 month before (t6), 2 weeks after (t7), and 6 months after (t9) re‐entry to the home country. At each wave, participants were invited by e‐mail to visit the project website, log in, and complete an online survey. Psychological and sociocultural adaptation to the home country and well‐being were measured at each of these timewaves. Crucially, however, adaptation was operationalized as *expected adaptation* pre‐return (t6) and as *experienced adaptation* at entry (t7) and 6 months after return (t9).

Participants needed to have completed the pre‐return survey (t6) and at least one of the critical surveys following re‐entry (t7 and t9), resulting in a final sample size of 1319. Samples vary slightly across analyses (*N*_*t7*_ = 1268, *N*_*t9*_ = 1139, *N*_*t7&t9*_ = 1088).

### Measures

Surveys were administered in 10 different languages (English, Chinese, French, German, Italian, Japanese, Portuguese, Spanish, Thai, and Turkish), covering those most commonly spoken among participants. Participants who had a different mother tongue (13.7%) were able to complete the surveys in the language of their choosing (English was chosen in 99% of cases). More than 20 different concepts were recorded through the online surveys, but here we concentrate on only those measures relevant to the present research questions. Questions were personalized to the participant with regard to the host and home culture.

#### Well‐being

Well‐being was operationalized through measures of satisfaction with life and stress. Both were administered at each wave (t1–t9), but only t6, t7, and t9 are analyzed here. The 5‐item Satisfaction with Life Scale (Diener et al., 1985) asked participants to indicate their agreement on a 7‐point scale (1 = *strongly disagree*, 7 = *strongly agree*) on items such as “on the whole, I am satisfied with myself.” Reliability was good at all waves (*α*'s > .80).

The short 4‐item version of the Perceived Stress Scale (Cohen et al., 1983) asked participants to indicate frequency on a 7‐point scale (1 = *never*, 7 = *always*) on items such as “In the last 2 weeks how often have you felt you were unable to control the important things in your life.” Reliability was good at all waves (*α*'s > .70).

#### Cultural adaptation

Two scales were used to assess sociocultural and psychological dimensions of cross‐cultural adaptation (Searle & Ward, 1990). Specifically, adaptation scales assessed sojourners' re‐entry expectations pre‐return (t6), as well as, experiences at re‐entry (t7) and 6 months after return (t9).

The 12‐item Brief Sociocultural Adaptation Scale (Demes & Geeraert, 2014) was adapted to measure sojourners' pre‐return expectations and re‐entry experiences concerning the ease of behavioral adaptation to social and cultural elements of the home country. The stem of the scale was subtly different at t6 (“Think about living in [name of the home country] again. How easy or difficult *do you think it will be* for you to adapt to …”), compared to t7 and t9 (“Think about living in [name of the home country] again. How easy or difficult *is it* for you to adapt to …”). Thus, participants indicated their expected/experienced difficulty using a 7‐point scale (1 = *very difficult*, 7 = *very easy*) on items such as “practicalities (getting around, using public transport, shopping),” and “social norms (how to behave in public, style of clothes, what people think is funny).” Reliability was good at all waves (*α*'s > .80).

The 8‐item Brief Psychological Adaptation Scale (Demes & Geeraert, 2014) was adapted to measure sojourners' expected (pre‐return) and experienced (post‐return) emotional and psychological aspects specific to a cultural relocation. Again, the stem of the questions was subtly different at t6 (“Think about living in [name of the home country] again. *How often do you think you will feel* …”) and t7 and t9 (“Think about living in [name of the home country] again. *In the last 2 weeks how often have you felt* …”). Thus, participants were asked to indicate their expected/experienced frequency on a 7‐point scale (1 = *never*, 7 = *always*) on items such as “excited about being in [name of the home country].” Reliability was good at all waves (*α*'s > .70).

### Data analytic strategy

Data analysis was conducted through a series of steps. First, the nature of the relation between expectations and experiences was examined through a series of correlations and mean comparisons to establish whether participants' experiences deviate from their expectations.

Next, we ran a total of 2 (well‐being measure: satisfaction with life and stress) × 2 (adaptation: psychological and sociocultural) × 2 (timewave: t7 and t9) = 8 polynomial regressions followed by response surface analyses. This analyses allows to test whether well‐being is highest when adaptation expectations and experience are congruent or match (accuracy hypothesis, prediction of ToME, see left panel of Figure 1) or whether well‐being is highest when experience are higher than expectations (directional hypothesis, prediction of the EVT, right panel of Figure 1). Compared to difference score methods, more information is retained through polynomial regression and reliability is higher (Edwards, 2002). We tested the following model (all predictors mean‐centered):

$$\text{well}‐\text{being}=b_{0}+b_{1}\text{Expectations}+b_{2}\text{Experience}+b_{3}{\text{Expectations}}^{2}+b_{4}\text{Expectations}*\text{Experience}+b_{5}{\text{Experience}}^{2}+e$$

A three‐dimensional plot of the data with all three variables (expectations, experience, and well‐being) can be followed up by an analysis of the plane, the Response Surface Analysis, which returns four RSA coefficients, *a*_*1*_ to *a*_*4*_. Each a function of two or more regression coefficients (Barranti et al., 2017), the RSA coefficients are defined as follows: *a*_*1*_ = *b*_*1*_ + *b*_*2*_ (the slope of the line of congruence), *a*_*2*_ = *b*_*3*_ + *b*_*4*_ (the curvature of the line of congruence), *a*_*3*_ = *b*_*1*_ − *b*_*2*_ (the slope of the line of incongruence), *a*_*4*_ = *b*_*3*_ − *b*_*4*_ + *b*_*5*_ (the curvature of the line of incongruence).

The coefficients *a*_*3*_ and *a*_*4*_ are most relevant for our research. A negative *a*_*3*_, for example, would imply that the plane is tilted downward to the right (similar to the plane in Figure 1, right panel). In contrast, a negative *a*_*4*_ would result in the saddle shape illustrated in the left panel of Figure 1 (see Barranti et al., 2017, for a visualization of the response surface analysis coefficients). There are several conditions that need to be fulfilled before it can be concluded that the data support a congruence effect (Humberg et al., 2019), including *a*_*3*_ ≈ 0 and *a*_*4*_ < 0.

The response surface analysis allows us to test the conflicting predictions of the ToME and the EVT. The saddle shape predicted by the ToME results from *a*_*4*_ < 0 (cf. Figure 1, left panel, where *a_4_* = −1, whereas all other coefficients are 0). In contrast, the main prediction of the EVT is that positive violations result in higher well‐being, which would imply that *a*_*3*_ < 0; that is, the plane has a negative slope from the top left to the bottom right corner, as illustrated in Figure 1, right panel. Note that the EVT does not rule out a (weaker) congruence effect (cf. Burgoon, 2015). Together, *a*_*3*_ ≈ 0 and *a*_*4*_ < 0 would support the ToME, whereas *a*_*3*_ < 0 would support the EVT, when the dependent variable is satisfaction with life. In contrast, when the dependent variable is perceived stress, which is negatively correlated with satisfaction with life (Table 1), *a*_*3*_ > 0 would support the EVT. We also tested whether including well‐being levels pre‐departure has an impact. Results were very similar (see Table S4).

**TABLE 1:** Mean, standard deviation, and bivariate correlations are shown for sociocultural adaptation, psychological adaptation, perceived stress, and satisfaction with life prior to return (t6), at re‐entry (t7) and 6 months after re‐entry (t9)

		M	SD	1	2	3	4	5	6	7	8	9	10	11	12
1	*Expected* sociocultural adaptation (t6)	5.09	1.05	—	.56**	.44**	.47**	.39**	.33**	−.15**	−.27**	−.21**	.08*	.22**	.18**
2	Sociocultural adaptation (t7)	5.19	1.05		—	.55**	.43**	.61**	.38**	−.09*	−.38**	−.21**	.04	.28**	.18**
3	Sociocultural adaptation (t9)	5.51	.92			—	.28**	.39**	.53**	−.16**	−.37**	−.35**	.14**	.32**	.35**
4	*Expected* psychological adaptation (t6)	4.15	.91				—	.66**	.48**	−.03	−.30**	−.15**	−.10**	.15**	.09*
5	Psychological adaptation (t7)	4.24	1.06					—	.57**	−.05	−.46**	−.23**	−.06	.28**	.15**
6	Psychological adaptation (t9)	4.30	.96						—	−.11**	−.35**	−.39**	.04	.20**	.30**
7	Perceived stress (t6)	2.68	1.00							—	.46**	.48**	−.55**	−.41**	−.35**
8	Perceived stress (t7)	2.83	1.03								—	.56**	−.28**	−.55**	−.39**
9	Perceived stress (t9)	2.90	1.06									—	−.33**	−.42**	−.60**
10	Satisfaction with life (t6)	5.46	1.05										—	.62**	.57**
11	Satisfaction with life (t7)	5.36	1.03											—	.68**
12	Satisfaction with life (t9)	5.24	1.09												—

*Note*: *p < .005, **p < .001.

A large sample size (*N* > 1000) greatly increases statistical power. Some scholars have argued that the a priori significance threshold (*α*) should be adjusted to minimize the overall chance of committing either a Type I or Type II error (see Mudge et al., 2012). For these reasons, the a priori significance level was adjusted to a more conservative level of *α* = .005 (see also Benjamin et al., 2018).

## RESULTS

Preliminary analyses were conducted across all variables. Descriptive statistics and bivariate correlations are reported in Table 1. Expected sociocultural and psychological adaptation were positively associated with experienced sociocultural and psychological adaptation as well as satisfaction with life, but negatively with stress.

### Return expectations versus experiences

To explore the relationship sojourner's adaptation expectations and experience, their bivariate correlations were examined. Expectations prior to re‐entry (t6) were positively and significantly correlated with re‐entry adaptation, for both sociocultural (t7: *r* = .56, *p* < .001, t9: *r* = .44, *p* < .001) and psychological adaptation (t7: *r* = .66, *p* < .001, t9: *r* = .48, *p* < .001). Thus, returnees who anticipated high adaptation on average reported experiencing higher levels of adaptation, both on re‐entry and 6 months after re‐entry.

Next, a repeated measures ANOVA was conducted to examine whether adaptation expectations and experience differed. Specifically, the analyses compared adaptation across timewaves (t6 vs. t7 vs. t9). For sociocultural adaptation, an effect of time emerged, *F*(2, 2046) = 112.70, *p* < .001. Follow‐up analyses showed that sociocultural adaptation 6 months after re‐entry (*M*_*t9*_ = 5.51, *SD* = .91) was higher than either pre‐return expectations (*M*_*t6*_ = 5.09, *SD* = 1.04, *p* < .001) or adaptation at re‐entry (*M*_*t7*_ = 5.14, *SD* = 1.05, *p* < .001). There was no difference between pre‐return expectations and adaptation at re‐entry (*p* = .096).

For psychological adaptation, a similar effect of time emerged, *F*(2, 2046) = 19.28, *p* < .001. Compared to re‐entry expectations (*M*_*t6*_ = 4.13, *SD* = .91), psychological adaptation was higher at re‐entry (*M*_*t7*_ = 4.20, *SD* = 1.06, *p* < .001) and half a year after re‐entry (*M*_*t9*_ = 4.30, *SD* = .95, *p* < .001). Interestingly, the difference between t7 and t9 was also significant (*p* < .001).

### Congruence and perceived stress

**FIGURE 2:**
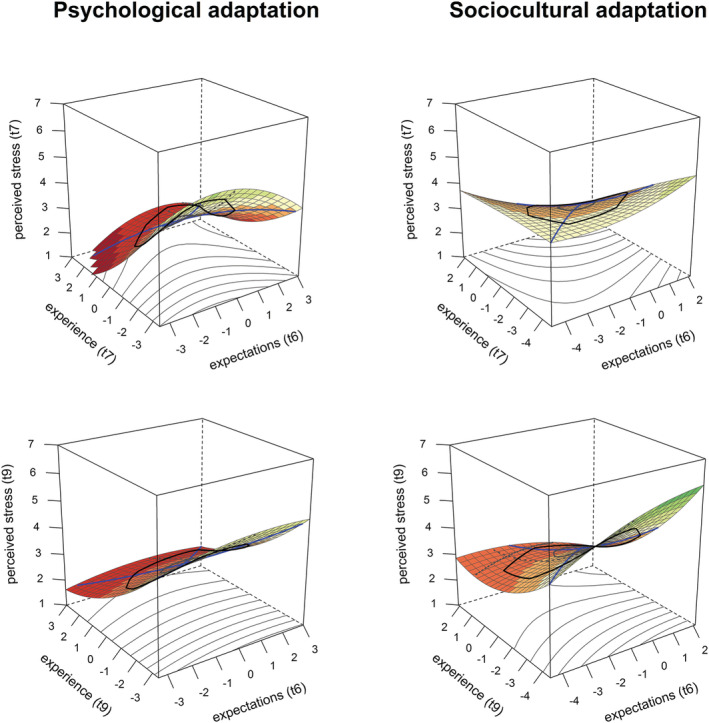
Three‐dimensional association between expected and experienced sociocultural (left panels) or psychology adaptation (right panels) with perceived stress at re‐entry (t7, top panels) and 6 months after re‐entry (t9, bottom panels)

As predicted by the EVT, stress is lowest if psychological and sociocultural adaptation experiences are better than expected across time points (t7 and t9). To test this, the data were plotted (see Figure 2). Visual inspection showed that the plane reached its lowest points (i.e. lowest stress levels) when experience was better than expected, and conversely reaches its highest points when expectations were more positive than experience. This observation was supported by significant positive *a*_*3*_‐coefficients (Table  2). More specifically, the effect was driven by a negative linear effect of experience *b*_*2*_ and a non‐significant linear effect of expectations *b*_*1*_ (recall that *a*_*3*_ = *b*_*1*_ − *b*_*2*_). In other words, whereas experience was negatively associated with stress (while controlling for expectations), expectations were unrelated to stress (while controlling for experience).

**TABLE 2:** Results of the polynomial regression and response surface analyses on perceived stress as a function of expectation and experience of adaptation (psychological or sociocultural) at re‐entry (t7) or 6 months after re‐entry (t9)

	Psychological adaptation		Sociocultural adaptation	
	t7	t9	t7	t9
Polynomial regression coefficients				
expectation (*b*_*1*_)	.01	.05	−.07	−.09
experience (*b*_*2*_)	−.45**	−.45**	−.34**	−.35**
expectation^2^ (*b*_*3*_)	−.09^†^	−.02	.03	−.03
expectation × experience (*b*_*4*_)	.05	−.01	−.06	−.07
experience^2^ (*b*_*5*_)	.06	.05	−.01	.07
Response surface analysis				
line of congruence slope (*a*_*1*_)	−.44**	−.40**	−.41**	−.43**
line of congruence curvature (*a*_*2*_)	.02	.02	−.03	−.03
line of incongruence slope (*a*_*3*_)	.46**	.50**	.27**	.26**
line of incongruence curvature (*a*_*4*_)	−.09	.03	.09	.10
Model statistics: *R* ^*2*^	.23**	.16**	.16**	.13**

*Note:* t7: at re‐entry; t9: 6 months after re‐entry, *b*_*1*_: expectation, *b*_*2*_: experience, *b*_*3*_: expectation (quadratic), *b*_*4*_: interaction term, *b*_*5*_: experience (quadratic), *a*_*1*_ = *b*_*1*_ + *b*_*2*_ (*Do matches at high values have different outcomes than matches at low values?*), *a*_*2*_ = *b*_*3*_ + *b*_*4*_ (*Do matches at extreme values have different outcomes than matches at less extreme values?*), *a*_*3*_ = *b*_*1*_ − *b*_*2*_ (*Is one mismatch [X > Y] better or worse than the other [X < Y]?*), *a*_*4*_ = *b*_*3*_ − *b*_*4*_ + *b*_*5*_ (*Are matches better or worse than mismatches?*), explanations for *a*_*1*_ − *a*_*4*_ are verbatim quotes from Barranti et al. (2017, p. 469).

^†^ *p* < .01.

* *p* < .005.

** *p* < .001.

In contrast, none of the *a*_*4*_‐coefficients were significant, which suggests that the match between expectations and experience is irrelevant for people's stress. This corresponds with the absence of the reversed saddle shape in Figure 2. Both a significant *a*_*4*_ coefficient and the reverse saddle shape would have been predicted by the accuracy hypothesis (ToME), yet these were not supported by the data.

Additionally, all *a*_*1*_‐coefficients were negative and significant (Table 2), which imply that stress is higher when both expectations and experiences reflect low levels of adaptation compared to when both are high (Figure 2). This finding is again driven by a negative linear effect of experience *b*_*2*_ and a non‐significant linear effect of expectations *b*_*1*_ (recall that *a*_*1*_ = *b*_*1*_ + *b*_*2*_). Although not directly relevant to either theory, these findings do shed light on the role of expectations and experience in well‐being.

### Congruence and satisfaction with life

**FIGURE 3:**
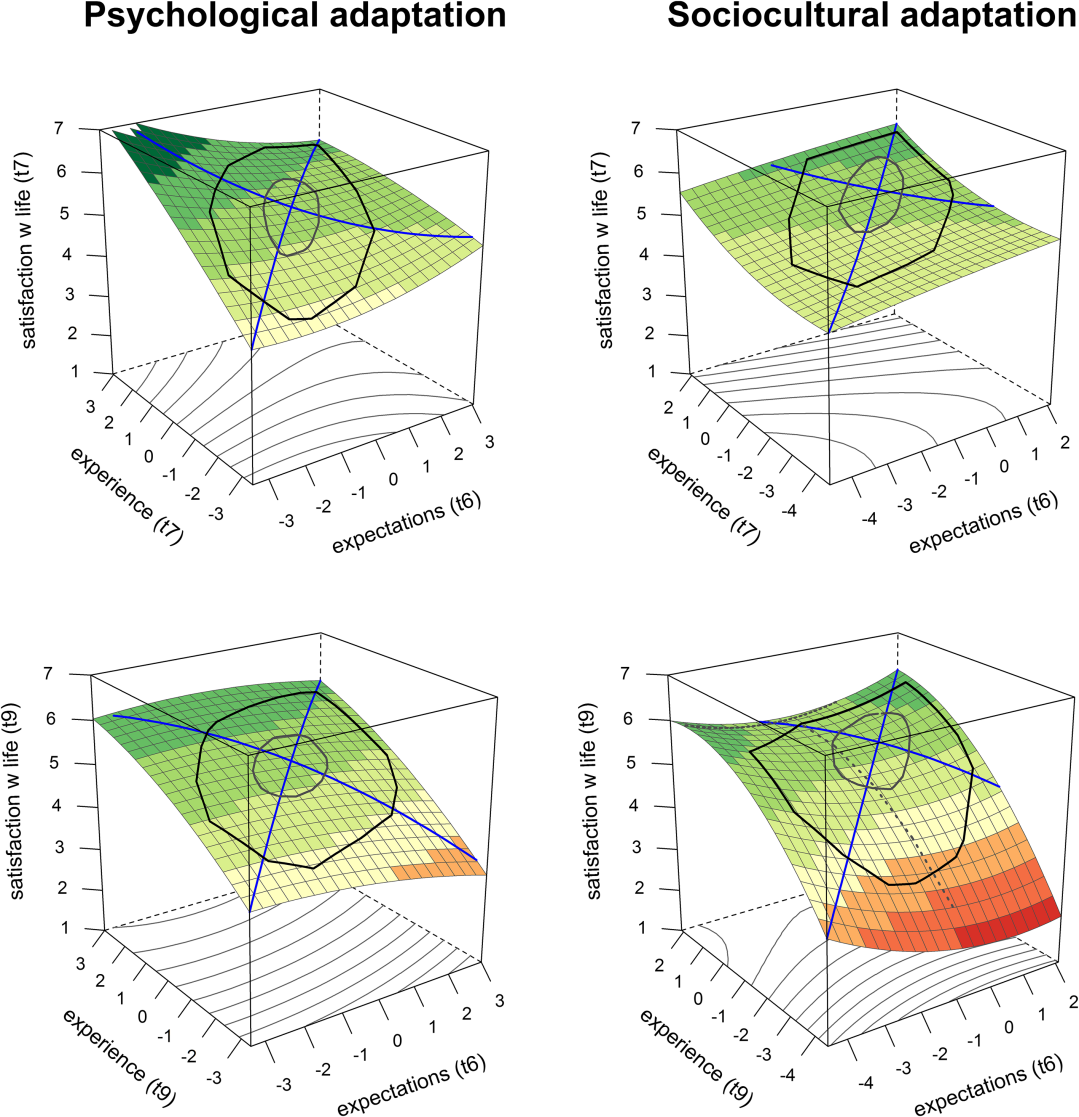
Three‐dimensional association between expected and experienced sociocultural (left panels) or psychology adaptation (right panels) with satisfaction with life at re‐entry (t7, top panels) and 6 months after re‐entry (t9, bottom panels)

The pattern for satisfaction with life was exactly the opposite (but conceptually identical). As predicted by the EVT, satisfaction with life is highest if experiences were better than expected across time points (t7 and t9) and types of adaptation (psychological and sociocultural). Visual inspection of the data (see Figure 3) showed that the plane reached its highest points (i.e. highest satisfaction with life levels) when experience was more favorable than expected, and conversely reaches its lowest points when expectations were more positive than experience. Conclusions from the visual examination were supported by significant negative *a*_*3*_‐coefficients (Table 3). Once more, the effect (recall that *a*_*3*_ = *b*_*1*_ − *b*_*2*_) was driven by a positive linear effect of experience (*b*_*2*_) and a non‐significant linear effect of expectations (*b*_*1*_). That is, experience is, when controlling for expectations, positively associated with satisfaction with life, whereas expectations, when controlling for experience, are unrelated to satisfaction with life.

**TABLE 3:** Results of the polynomial regression and response surface analyses on satisfaction with life as a function of expectation and experience of adaptation (psychological or sociocultural) at re‐entry (t7) or 6 months after re‐entry (t9)

	Psychological adaptation		Sociocultural adaptation	
	t7	t9	t7	t9
Polynomial regression coefficients				
expectation (*b*_*1*_)	−.07	−.08	.08	.07
experience (*b*_*2*_)	.31**	.38**	.25**	.35**
expectation^2^ (*b*_*3*_)	.03	−.02	−.00	.05
expectation x experience (*b*_*4*_)	−.06	.02	−.00	.03
experience^2^ (*b*_*5*_)	−.01	−.03	.03	−.08
Response surface analysis				
line of congruence slope (*a*_*1*_)	.24**	.29**	.33**	.42**
line of congruence curvature (*a*_*2*_)	−.04	−.04	.03	−.00
line of incongruence slope (*a*_*3*_)	−.38**	−.46**	−.17*	−.28**
line of incongruence curvature (*a*_*4*_)	.08	−.07	.03	−.06
Model statistics: *R* ^*2*^	.09**	.10**	.09**	.13**

*Note:* t7: at re‐entry; t9: 6 months after re‐entry, *b*_*1*_: expectation, *b*_*2*_: experience, *b*_*3*_: expectation (quadratic), *b*_*4*_: interaction term, *b*_*5*_: experience (quadratic), *a*_*1*_ = *b*_*1*_ + *b*_*2*_ (*Do matches at high values have different outcomes than matches at low values?*), *a*_*2*_ = *b*_*3*_ + *b*_*4*_ (*Do matches at extreme values have different outcomes than matches at less extreme values?*), *a*_*3*_ = *b*_*1*_ − *b*_*2*_ (*Is one mismatch [X > Y] better or worse than the other [X < Y]?*), *a*_*4*_ = *b*_*3*_ − *b*_*4*_ + *b*_*5*_ (*Are matches better or worse than mismatches?*), explanations for *a*_*1*_–*a*_*4*_ are verbatim quotes from Barranti et al. (2017, p. 469).

^†^ *p* < .01.

* *p* < .005.

** *p* < .001.

In contrast, none of the *a*_*4*_ coefficients were significant, which suggests that whether expectations and experience match is irrelevant for people's satisfaction with life. In addition, the saddle shape was not visible on the graphs (Figure 3). Thus as a set, the data do not support the accuracy hypothesis (ToME).

Additionally, we found that all *a*_*1*_‐coefficients were significantly positive (Table 3), which imply that satisfaction with life is lower when both expectations and experiences reflect low levels of adaptation compared to when both are high (Figure 3). This finding is again driven by a positive linear effect of experience *b*_*2*_ and a non‐significant linear effect of expectations *b*_*1*_ (recall that *a*_*1*_ = *b*_*1*_ + *b*_*2*_).

In sum, across eight analyses the *a*_*3*_ coefficient (the slope associated with the line of incongruence) was significant, providing strong support for the directional hypothesis (i.e. EVT). In contrast, the *a*_*4*_ coefficient (the curvature of the line of incongruence) was not significant for either stress or satisfaction with life.

### Sensitivity analyses

To ensure the robustness of these findings, a series of additional analyses were conducted. First, the return data were analyzed through an alternative method, which decomposes the discrepancy between expectation and experience into a magnitude and direction component (see supporting information). This analysis provided consistent support for a directional hypothesis for stress at both timewaves. In particular, the results show that the adaptation discrepancy magnitude is negative associated with stress, but only when the expectations were undermet. Results for satisfaction with life were conceptually similar. When the experience was relatively similar to the expectation (i.e. small mismatches), the direction of the mismatch matters not; however, as the discrepancy increases, the pattern changes. Sojourners reported positive well‐being when the experience was better than expected, but lower well‐being when the experience failed to match expectations.

Second, additional analyses were conducted controlling for participants' age and sex. There was no association between age (at t1) and our measured variables (all *p*'s > .20), so we did not consider age any further. For sex, males reported higher levels of psychological adaptation (*p*'s < .005 at t6, t7, and t9) and sociocultural adaptation (*p*'s < .005 at t6, t7, and t9), and lower levels of stress at t6 (*p* = .007) and t7 (*p* < .001). Given these effects, the original polynomial regression and response surface analyses were repeated controlling for sex. As expected, following the results from the bivariate correlation, sex was a significant predictor for stress at entry (t7). Importantly, however, the addition of the control variable did not alter the pattern of results in any of the analyses, even when including satisfaction with life or stress before entry (t6) as an additional control variable (see supporting information).

## DISCUSSION

The relationship between pre‐return expectations and experiences of return adaptation after a year abroad was examined using data from a longitudinal sojourner project. A positive correlation between adaptation expectations and actual experience upon re‐entry and 6 months later indicated that sojourners had a relatively good understanding of the trajectory ahead. At the mean level, sojourners reported comparatively higher adaptation than anticipated, which was more pronounced in the later timewave. Scholars previously discussed the difficulties associated with return adaptation, which often come as a surprise to returnees (Arthur, 2003; Gaw, 2000; Young, 2014). Yet, it seems our sojourners were reasonably well aware of the return challenges. This does not need to come as a surprise, as AFS Intercultural programs is known for the level of preparation of its exchange students in all aspects of their educational sojourn, including the return.

Turning our attention to the impact of expectation‐experience discrepancies, two theoretical accounts emerge. The ToME postulates that greater congruence between expectations and experience will generally be associated with more positive outcomes (Caligiuri et al., 2001; Porter & Steers, 1973; Wanous, 1992; Wanous et al., 1992). In contrast, the EVT states that negative, but not positive, discrepancies will be associated with greater psychological distress (Burgoon, 1995, 2015). Crucially, previous empirical support for ToME (Black, 1992) and EVT (Rogers & Ward, 1993) was inconclusive due to methodological concerns.

To test the impact of the expected‐experienced adaptation incongruities on stress and well‐being, polynomial regression and response surface analyses were conducted (Barranti et al., 2017; Humberg et al., 2019), with stress and satisfaction with life as dependent variables. This approach is more robust compared to alternative approaches such as difference scores (Edwards, 2002). If experience was more positive than expected, stress was lower and satisfaction was higher, compared to when experiences were more negative than expected. Similarly, when expectations and experience were both low, stress was higher and satisfaction with life was lower compared to when both expectations and experience were high. This effect was driven by a negative (positive) linear association between experience and stress (satisfaction with life). Taken together, these results are clearly consistent with the directional effect of re‐entry expectations as postulated by the EVT. In contrast, we found no support for the ToME. When expectations and experience matched did not seem to make stress lower or satisfaction with life higher.

Compared to the paucity of research on return expectations, there has been more research on expectations‐experience discrepancies on entry into the host or settlement culture. Findings have been mixed, possibly due to the many different methodological approaches used. However, a series of recent studies using robust methods, have provided at least partial support for a directional effect of entry expectations (Geeraert et al., 2021; Mähönen & Jasinskaja‐Lahti, 2013). Interestingly, this would suggest that a similar process may be at play during entry and return phases of prolonged intercultural travel.

Our research clearly demonstrates that overly optimistic expectations, which are discrepant from actual re‐entry experiences, lead to increased stress and lower levels of life satisfaction. Nevertheless, on average participants in this study reported that re‐entry adaptation was easier than anticipated. These findings diverge from related research with international students and expatriates; studies have shown that sojourners' pre‐return expectations are more likely to be met with no significant differences between expectations and experiences (Rogers & Ward, 1993) or undermet with experiences being less favorable than expected (Stroh et al., 1998). Notably, Kraimer et al.'s (2016) review of four decades of research on repatriation concluded that re‐entry is “often” associated with unmet expectations (p. 95). Moreover, unmet expectations negatively impact both subjective well‐being in returnees and a variety of employment‐related outcomes, including expatriate job performance and retention. A critical question, then, is, how can undermet expectations be avoided?

We suggest that intercultural training can address this question. It has been widely noted that psychological distress during re‐entry is typically predicated on the unexpectedness of re‐entry problems (Gaw, 2000; Martin, 1993; Young, 2014). Researchers and practitioners alike have highlighted the importance of preparation for re‐entry, including the need to set expectations for repatriation (Martin & Harrell, 2004; Sussman, 1986). AFS, who hosted the intercultural exchange students in our research, is particularly effective in achieving this objective and offer re‐entry orientations as well as welcome home events and a global link for returnees. More generally, Martin and Harrell (2004) claim repatriation training is essential for successful re‐entry and have described some established training programs developed for international students and professionals. There is also a variety of online resources, some freely available from universities and international organisations, as well as programs that can be purchased from global corporations. Importantly, education providers that offer study abroad programs as well as multinational companies that rely on overseas assignments have a role to play in equipping cross‐cultural travelers with the best tools to succeed in both outward‐ and inward‐bound cultural transitions.

### Strengths and limitations

The present study has a number of notable strengths. The impact of sojourner's return expectations on well‐being had not been previously examined with the same methodological rigor. The use of longitudinal data allowed us to compare adaptation expectations pre‐return with adaptation experiences post‐return. Thus, the study did not rely on methodologically flawed retrospective expectations used in other research (Black, 1992). The large sample size and broad geographical spread of sending and hosting countries is an additional strength, addressing shortcomings from previous findings relying on samples that were either small or had limited geographical spread (Black, 1992; Rogers & Ward, 1993). Furthermore, the findings were shown to be robust across time, types of adaptation, and outcome measures.

The association between the expectation‐experience discrepancy and well‐being is consistent across all analyses. Subsequent analyses (see supporting information) controlled for baseline well‐being (prior to return) in each analysis, with an identical pattern of results. Controlling for baseline, the outcome can perhaps best be interpreted as a change in well‐being from baseline. Thus, the expectation‐experience discrepancy is essentially associated with a change in well‐being. In other words, under conditions of a negative mismatch, a larger discrepancy was associated with a drop in well‐being. Nonetheless, the current analyses do prevent us from drawing causal inferences. According to Expectation Violation Theory, negative mismatches will lead to lower well‐being. However, different causal explanations can easily be construed. For instance, sojourners who experience lower well‐being may be more likely to perceive or recall adaptation challenges, causing them to rate their experienced adaptation less favorable. Alternatively, the association between the two variables may be caused by a third variable. Future research may seek to address this limitation by relying on different methods and data analytic strategies, such as experimental designs.

A further limitation is that these data are based on a sojourner sample of exchange students, and thus it is less clear to what extent the current findings would generalize to other samples such as returning migrants or expatriates. However, until future research addresses this gap, we can only speculate that the pattern of results will be similar across diverse acculturating groups.

### Conclusion

Using longitudinal data from a large and diverse sample, this study examined the impact of return expectations. In particular, experiences that were progressively worse than expected had negative consequences for stress and well‐being. This pattern was consistent with the directional effect as proposed by the EVT (Burgoon, 1995, 2015), thereby mimicking similar patterns that were reported for entry expectations. Taken together, these findings suggest that returning home is an integral part of the sojourn experience.

## CONFLICT OF INTEREST

All authors declare that they have no conflicts of interest.

## ETHICS STATEMENT

Ethics for the study was granted by the University of Essex' Ethics Committee (id NG0802).

## Supporting information


**Table S1.** Bivariate correlations are shown between the raw adaptation scores (at t6, t7, t9) and the magnitude, direction and interactions scores at re‐entry (t7) and 6 months after re‐entry (t9). Sociocultural adaptation scores are presented above the axis. Psychological adaptation scores are presented below the axis.
**Table S2.** The effect of the expectation ‐ experience mismatch on perceived stress. Results of four moderated regression analyses are shown, examining the role of magnitude and direction, for sociocultural and psychological adaptation, at re‐entry (t7) and 6 months after re‐entry (t9). Betas, significance levels, and summary statistics are provided for each analysis.
**Table S3.** The effect of the expectation ‐ experience mismatch on satisfaction with life. Results of four moderated regression analyses are shown, examining the role of magnitude and direction, for sociocultural and psychological adaptation, at re‐entry (t7) and 6 months after re‐entry (t9). Betas, significance levels, and summary statistics are provided for each analysis.
**Table S4.** Results of the polynomial regression and response surface analyses with the difference score of well‐being after re‐entry minus well‐being before re‐entry as dependent variable.
**Figure S1.** Simple slope analyses showing levels of perceived stress at re‐entry (t7, top panels) and 6 months after re‐entry (t9, bottom panels) as a function of magnitude (small to large) and direction (negative vs positive mismatch) of the discrepancy between re‐entry expectations (t6) and experience of sociocultural (left panels) and psychological adaptation (right panels)
**Figure S2.** Simple slope analyses showing levels of satisfaction with life at re‐entry (t7, top panels) and 6 months after re‐entry (t9, bottom panels) as a function of magnitude (small to large) and direction (negative vs positive mismatch) of the discrepancy between re‐entry expectations (t6) and experience of sociocultural (left panels) and psychological adaptation (right panels)
**Figure S3.** Three‐dimensional association between expected and experienced sociocultural (left panels) or psychology adaptation (right panels) with perceived stress at re‐entry (t7, top panels) and 6 months after re‐entry (t9, bottom panels), controlling for perceived stress at t6.
**Figure S4.** Three‐dimensional association between expected and experienced sociocultural (left panels) or psychology adaptation (right panels) with satisfaction with life at re‐entry (t7, top panels) and 6 months after re‐entry (t9, bottom panels), controlling for satisfaction with life at t6.

## Data Availability

The datasets generated and/or analyzed during the current study are not publicly available but are available from the corresponding authors on reasonable request.

## ENDNOTES

^1^
The ESRC funded project was created to examine different aspects of the acculturation process. Earlier papers have looked at stress trajectories (Demes & Geeraert, 2015) and the impact of looseness–tightness on adaptation (Geeraert et al., 2019). The current study on expectations has a different focus, yet, like the other topics, was planned at the design stage.

^2^
AFS (originally the American Field Service) begun as a service of volunteer ambulance drivers in 1914; it has since evolved into an international youth exchange organization.
