# Supplementary material for: Returning home: The role of expectations in re‐entry adaptation
Source: Appl Psychol Health Well Being. 2022 Apr 5;14(3):949–66. doi: 10.1111/aphw.12361 (PMC9541004; doi:10.1111/aphw.12361)
Supplement: Supplementary file 1 — Table S1. Bivariate correlations are shown between the raw adaptation scores (at t6, t7, t9) and the magnitude, direction and interactions scores at re‐entry (t7) and 6 months after re‐entry (t9). Sociocultural adaptation scores are presented above the axis. Psychological adaptation scores are presented below the axis. Table S2. The effect of the expectation ‐ experience mismatch on perceived stress. Results of four moderated regression analyses are shown, examining the role of magnitude and direction, for sociocultural and psychological adaptation, at re‐entry (t7) and 6 months after re‐entry (t9). Betas, significance levels, and summary statistics are provided for each analysis. Table S3. The effect of the expectation ‐ experience mismatch on satisfaction with life. Results of four moderated regression analyses are shown, examining the role of magnitude and direction, for sociocultural and psychological adaptation, at re‐entry (t7) and 6 months after re‐entry (t9). Betas, significance levels, and summary statistics are provided for each analysis. Table S4. Results of the polynomial regression and response surface analyses with the difference score of well‐being after re‐entry minus well‐being before re‐entry as dependent variable. Figure S1. Simple slope analyses showing levels of perceived stress at re‐entry (t7, top panels) and 6 months after re‐entry (t9, bottom panels) as a function of magnitude (small to large) and direction (negative vs positive mismatch) of the discrepancy between re‐entry expectations (t6) and experience of sociocultural (left panels) and psychological adaptation (right panels) Figure S2. Simple slope analyses showing levels of satisfaction with life at re‐entry (t7, top panels) and 6 months after re‐entry (t9, bottom panels) as a function of magnitude (small to large) and direction (negative vs positive mismatch) of the discrepancy between re‐entry expectations (t6) and experience of sociocultural (left panels) and psychological adaptatio [file APHW-14-949-s001.docx]

**Supplementary Materials**

Returning Home: The Role of Expectations on Re-entry Adaptation

Geeraert, N., Ward, C., & Hanel, P.

**Discrepancy analyses**

The accuracy and directional hypotheses were examined by assessing whether expectation-experience discrepancies influence students’ well-being at the point of re-entry and six months later. Statistically, the components of the discrepancy were operationalized as the absolute difference score (for magnitude) and as a simple contrast (to indicate direction). An accuracy effect (supporting ToME) would be evident from an association between higher discrepancy with lower well-being, regardless of direction. In terms of its components, an effect of magnitude, in the absence of an effect of direction would provide support for the accuracy hypothesis. In contrast, a directional effect (supporting EVT) would be evident from an association between larger discrepancy and poorer well-being, but only when the experience is worse than expected (i.e. a negative mismatch). Thus, an interaction between magnitude and direction would be consistent with the directional hypothesis.

**Discrepancy components**

In preparation for the analyses, the discrepancy between sojourners’ expected and experienced adaptation was broken down into a magnitude and a direction component. For each type of adaptation (psychological and sociocultural) and for both time points (t7 and t9), adaptation was regressed on adaptation expectations (t6). The standardized residuals resulting from these analyses were saved to represent the difference between sojourners’ return adaptation and the predicted values given their re-entry expectations. The residuals were used to compute *magnitude* (mean centered absolute score) and *direction* (contrast coded as 1 for a positive mismatch, and -1 for a negative mismatch) for both types of adaptation. Finally, bivariate correlations between the original adaptation measures and its components were inspected (see Table S1).

**Implications for Perceived Stress**

Using a series of moderated regressions, returnee’s stress was regressed on both magnitude and direction of the expectation-experience discrepancy. In each of the analyses, the following steps were conducted. First, the pre-return baseline (t6) of stress was entered. In the second step, the centered variables of magnitude and direction were added. Finally, the product of magnitude and direction was added. In terms of multicollinearity, none of the predictors were strongly correlated (all *r*’s < .30, see Table S1), and an inspection of the multicollinearity statistics suggested no issues in any of the regressions (all Tolerances > .90, all VIF’s < 1.05). Separate analyses were performed for both types of adaptation (sociocultural and psychological) and for both timewaves (t7 and t9) resulting in 4 analyses (see Table S2).

***Sociocultural Adaptation.***

Stress at entry (t7) was regressed on the baseline (stress at t6). Accounting for 22% of the variance, baseline stress was positively associated with stress at entry. In the second step, the magnitude and direction components of the sociocultural adaptation discrepancy were added, accounting for an additional 6% of the variance. Both components were significant predictors. Higher levels of stress were more strongly associated with larger discrepancies (a positive effect of magnitude) and a negative mismatch (a negative effect of direction). The addition of the interaction of magnitude x direction was also significant (β = -.15, p < .001). The interaction was subsequently decomposed by means of a simple slope analyses (see Figure S1, top left panel). The analysis showed that magnitude was associated with higher levels of stress (controlling for baseline) when the mismatch was negative (*B* = .37, *SE* = .07, *p* < .001), but not when it was positive (*B* = -.13, *SE* = .06, *p* = .03).

The same analysis was conducted 6 months after return. Controlling for baseline, stress at t9 was associated negatively with direction and positively with magnitude. However, the interaction of magnitude x direction qualified these effects (β = -.12, *p* < .001). Simple slope analyses (see Figure S1, bottom left graph) revealed that the degree of discrepancy was associated with higher stress when expectations were undermet negative (*B* = .36, *SE* = .07, *p* < .001), but not when expectations were overmet (*B* = -.07, *SE* = .07, *p* = .29).

***Psychological Adaptation***

Next, the regression analyses for stress were repeated for psychological adaptation. Stress (at t7) was negatively related to direction and positively to magnitude. Importantly, the magnitude x direction interaction for psychological adaptation was also significant (β = -.21, *p* < .001). The simple slopes (see Figure S1, top right graph) suggested that larger discrepancies were associated with lower levels of stress for positive mismatches (*B* = -.19, *SE* = .05, *p* < .001), but higher levels for negative mismatches (*B* = .50, *SE* = .07, *p* < .001).

An identical pattern emerged at t9 (β = -.21, *p* < .001). Simple slopes analyses (see Figure S1, bottom right graph) revealed that larger discrepancies were associated with lower stress when the mismatch was positive (*B* = -.34, *SE* = .06, *p* < .001), but higher stress when the mismatch was negative (*B* = .45, *SE* = .07, *p* < .001).

In sum, analyses consistently support the directional hypothesis for stress at re-entry and 6 months after return. In particular, the results show that the adaptation discrepancy magnitude is negative associated with stress, but only when the expectations were undermet.

**Implications for satisfaction with life**

The same series of analyses was repeated for satisfaction with life at re-entry (t7) and approximately 6 months after re-entry (t9). Separate analyses were performed for both types of adaptation and across both timewaves (see Table S3). Multicollinearity was not an issue in any of the analyses (*r*’s < .30, Tolerances > .90, VIF’s < 1.10).

***Sociocultural Adaptation***

Satisfaction with life (t7) was first regressed on the baseline (satisfaction with life at t6), accounting for 38% of the variance. Next, components of the discrepancy in sociocultural adaption were added. Satisfaction with life was independently associated with direction (β = .15, *p* < .001), but not with magnitude. More importantly, the interaction term was again significant (β = .14, *p* < .001). Simple slopes analyses (see Figure S2, top left graph), revealed the pattern of results to be conceptually identical (but reversed) compared to the analyses for stress. Specifically, a greater expectation-experience discrepancy was associated with higher levels of well-being (compared to the baseline) for positive mismatches (*B* = .22, *SE* = .05, *p* < .001), but lower well-being for negative mismatch (*B* = -.24, *SE* = .06, *p* < .001).

For satisfaction with life six months after return (t9), the interaction was again significant (β = .12, *p* < .001). Simple slopes analyses (see Figure S2, bottom left graph), revealed that a larger expectation-experience discrepancy was associated with lower well-being for undermet expectations (B = -.39, SE = .07, p < .001), but not for overmet expectations (B = .04, SE = .07, p = .58).

***Psychological Adaptation***

The regression of satisfaction with life on psychological adaptation revealed a similar pattern. Direction was a significant predictor (β = .20, *p* < .001), but this effect was qualified by a significant interaction (β = .12, *p* < .001). Simple slopes analyses (see Figure S2, top right graph) showed that, when controlling for baseline, the relationship between satisfaction with life and size of the discrepancy was positive when expectations were overmet (*B* = .14, *SE* = .04, *p* < .001), but negative when expectations were undermet (*B* = -.27, *SE* = .06, *p* < .001).

An identical pattern of results emerged for t9. Simple slopes analyses (see Figure S2, bottom right graph) suggested that the relationship between level of well-being and magnitude was positive when expectations were overmet (*B* = .26, *SE* = .07, *p* < .001), but negative when expectations were undermet (*B* = -.30, *SE* = .07, *p* < .001).

Across four analyses, results suggest that expectation-experience discrepancies upon returning home may have a directional effect on well-being. When the experience is relatively similar to the expectation (i.e. small mismatches), the direction of the mismatch matters not. However, as the discrepancy increases the pattern changes. Sojourners reported positive well-being when the experience was better than expected, but lower well-being when the experience failed to match expectations.

**Response Surface Analysis Controlling for Baseline**

To test the robustness of our findings, Response Surface Analyses were repeated controlling for t6 baseline. Specifically, tor each outcome variable (perceived stress and satisfaction with life) and for each timewave (t7 and t9) a difference score was created by subtracting the respective t6 baseline for each outcome variable. This resulted in 4 change scores (2 variable x 2 timewaves), which were then regressed on expected and experienced psychological and sociocultural adaptation using polynomial regression. This was followed by Response Surface Analysis.

The results largely replicate the original analyses, thereby support the EVT. Again, the response surface coefficient a3 was significant for all 8 analyses whereas a4 was non-significant (Table S4). For stress, the three-dimensional associations between expected and experienced psychological adaptations was plotted (Figure S3). Visual inspection of the figures for perceived stress clearly show that the saddle shape, predicted by ToME, is missing. A similar, but inverse, pattern emerges for satisfaction with life (Figure S4), which is in line with the analyses reported in the main manuscript.

**Table S1**. Bivariate correlations are shown between the raw adaptation scores (at t6, t7, t9) and the magnitude, direction and interactions scores at re-entry (t7) and 6 months after re-entry (t9). Sociocultural adaptation scores are presented above the axis. Psychological adaptation scores are presented below the axis.

|  |  | 1 | 2 | 3 | 4 | 5 | 6 | 7 | 8 | 9 |
| --- | --- | --- | --- | --- | --- | --- | --- | --- | --- | --- |
| 1 | t6 adaptation (raw score) | - | .56** | .44** | -.10** | .09* | -.11** | -.14** | .06 | -.09* |
| 2 | t7 adaptation (raw score) | .66** | - | .55** | -.16** | .70** | .46** | -.17** | .33** | .11** |
| 3 | t9 adaptation (raw score) | .48** | .57** | - | -.07 | .35** | .16** | -.28** | .75** | .51** |
| 4 | t7 magnitude | -.05 | -.03 | -.07 | - | -.08* | -.11** | .20** | -.04 | -.06 |
| 5 | t7 direction | .07 | .64** | .31** | -.04 | - | .01 | -.07 | .34** | .11** |
| 6 | t7 magnitude x direction | -.09* | .41** | .17** | .05 | .00 | - | -.10* | .16** | .17** |
| 7 | t9 magnitude | .02 | .01 | -.13** | .22** | -.01 | -.02 | - | -.11** | -.26** |
| 8 | t9 direction | -.01 | .22** | .67** | -.06 | .26** | .17** | -.08 | - | .01 |
| 9 | t9 magnitude x direction | .01 | .18** | .57** | -.02 | .18** | .16** | -.15** | .01 | - |

**Table S2.** The effect of the expectation - experience mismatch on perceived stress. Results of four moderated regression analyses are shown, examining the role of magnitude and direction, for sociocultural and psychological adaptation, at re-entry (t7) and 6 months after re-entry (t9). Betas, significance levels, and summary statistics are provided for each analysis.

|  | sociocultural adaptation | | | | | | |  | psychological adaptation | | | | | | |
| --- | --- | --- | --- | --- | --- | --- | --- | --- | --- | --- | --- | --- | --- | --- | --- |
|  | at entry (t7) | | |  | 6 months after re-entry (t9) | | |  | at entry (t7) | | |  | 6 months after re-entry (t9) | | |
|  | 1 | 2 | 3 |  | 1 | 2 | 3 |  | 1 | 2 | 3 |  | 1 | 2 | 3 |
| baseline | .47** | .46** | .46** |  | .49** | .46** | .46** |  | .47** | .46** | .46** |  | .49** | .47** | .45** |
| magnitude |  | .09** | .08* |  |  | .11** | .08* |  |  | .08** | .09** |  |  | .07^✝^ | .03 |
| direction |  | -.22** | -.22** |  |  | -.18** | -.18** |  |  | -.26** | -.26** |  |  | -.20** | -.21** |
| interaction |  |  | -.15** |  |  |  | -.12** |  |  |  | -.21** |  |  |  | -.24** |
| *R^2^* | .22 | .28 | .30 |  | .24 | .28 | .29 |  | .22 | .29 | .34 |  | .24 | .28 | .34 |
| *F* | 349.94** | 159.52** | 133.77** |  | 331.45** | 142.23** | 114.00** |  | 346.94** | 175.14** | 159.58** |  | 331.45** | 141.38** | 136.49** |
| *df* | 1, 1261 | 3, 1259 | 4, 1258 |  | 1, 1078 | 3, 1076 | 4, 1075 |  | 1, 1261 | 3, 1259 | 4, 1258 |  | 1, 1078 | 3, 1076 | 4, 1075 |
| *ΔR^2^* |  | .06 | .02 |  |  | .05 | .01 |  |  | .08 | .04 |  |  | .05 | .05 |
| *ΔF* |  | 51.83** | 41.24** |  |  | 36.65** | 21.28** |  |  | 70.20** | 79.95** |  |  | 35.69** | 87.64** |
| *Δdf* |  | 2, 1259 | 1, 1258 |  |  | 2, 1076 | 1, 1075 |  |  | 2, 1259 | 1, 1258 |  |  | 2, 1076 | 1, 1075 |

*Note.* ^✝^ *p* < .01, * *p* < .005, ** *p* < .001

**Table S3.** The effect of the expectation - experience mismatch on satisfaction with life. Results of four moderated regression analyses are shown, examining the role of magnitude and direction, for sociocultural and psychological adaptation, at re-entry (t7) and 6 months after re-entry (t9). Betas, significance levels, and summary statistics are provided for each analysis.

|  | sociocultural adaptation | | | | | | |  | psychological adaptation | | | | | | |
| --- | --- | --- | --- | --- | --- | --- | --- | --- | --- | --- | --- | --- | --- | --- | --- |
|  | at entry (t7) | | |  | 6 months after re-entry (t9) | | |  | at entry (t7) | | |  | 6 months after re-entry (t9) | | |
|  | 1 | 2 | 3 |  | 1 | 2 | 3 |  | 1 | 2 | 3 |  | 1 | 2 | 3 |
| baseline | .62** | .62** | .62** |  | .57** | .54** | .54** |  | .62** | .62** | .62** |  | .57** | .56** | .55** |
| magnitude |  | -.02 | -.01 |  |  | -.13** | -.10** |  |  | -.04 | -.04 |  |  | -.04 | -.01 |
| direction |  | .15** | .15** |  |  | .17** | .17** |  |  | .20** | .20** |  |  | .18** | .19** |
| interaction |  |  | .14** |  |  |  | .12** |  |  |  | .12** |  |  |  | .16** |
| *R^2^* | .38 | .41 | .43 |  | .32 | .37 | .39 |  | .38 | .42 | .44 |  | .32 | .36 | .39 |
| *F* | 786.42** | 286.84** | 232.43** |  | 517.65** | 212.71** | 168.04** |  | 786.42** | 311.58** | 248.41** |  | 517.65** | 202.10** | 169.23** |
| *df* | 1, 1261 | 3, 1259 | 4, 1258 |  | 1, 1078 | 3, 1076 | 4, 1075 |  | 1, 1261 | 3, 1259 | 4, 1258 |  | 1, 1078 | 3, 1076 | 4, 1075 |
| *ΔR^2^* |  | .02 | .02 |  |  | .05 | .01 |  |  | .04 | .02 |  |  | .04 | .03 |
| *ΔF* |  | 23.21** | 41.50** |  |  | 41.02** | 21.73** |  |  | 46.06** | 34.22** |  |  | 30.27** | 45.54** |
| *Δdf* |  | 2, 1259 | 1, 1258 |  |  | 2, 1076 | 1, 1075 |  |  | 2, 1259 | 1, 1258 |  |  | 2, 1076 | 1, 1075 |

*Note.* ^✝^ *p* < .01, * *p* < .005, ** *p* < .001

**Table S4.** Results of the polynomial regression and response surface analyses with the difference score of well-being after re-entry minus well-being before re-entry as dependent variable.

|  | change in perceived stress | | | | |  | change in satisfaction with life | | | | |
| --- | --- | --- | --- | --- | --- | --- | --- | --- | --- | --- | --- |
|  | psych adap | |  | sociocult adap | |  | psych adap | |  | sociocult adap | |
|  | t7 | t9 |  | t7 | t9 |  | t7 | t9 |  | t7 | t9 |
| Polynomial regression coefficients |  |  |  |  |  |  |  |  |  |  |  |
| expectation (b_1_) | .01 | .01 |  | .06 | .01 |  | .07 | .07 |  | -.02 | .03 |
| experience (b_2_) | -.40** | -.30** |  | -.31** | -.20** |  | .29** | .26** |  | .25** | .20** |
| expectation^2^ (b_3_) | -.05 | .02 |  | .03 | -.02 |  | .05 | -.02 |  | -.03 | -.01 |
| expectation x experience (b_4_) | -.05 | -.02 |  | -.04 | -.02 |  | -.02 | .00 |  | -.03 | .03 |
| experience^2^ (b_5_) | .15** | .09 |  | .05 | .09 |  | -.08 | -.06 |  | .01 | -.08 |
| Response surface analysis |  |  |  |  |  |  |  |  |  |  |  |
| line of congruence slope (a_1_) | -.39** | -.29** |  | -.25** | -.19** |  | .36** | .33** |  | .23** | .23** |
| line of congruence curvature (a_2_) | .04 | .09 |  | .03 | .05 |  | -.06 | -.07 |  | -.06 | -.06 |
| line of incongruence slope (a_3_) | .40** | .31** |  | .37** | .20* |  | -.22** | -.19** |  | -.26** | -.18^✝^ |
| line of incongruence curvature (a_4_) | .15 | .12 |  | .11 | .09 |  | -.02 | -.08 |  | .01 | -.12 |
| Model statistics: *R^2^* | .19** | .10** |  | .09** | .05** |  | .17** | .10** |  | .09** | .06** |

*Note.* ^✝^ *p* < .01, * *p* < .005, ** *p* < .001. psych adap: psychological adaptation, sociocult adap: sociocultural adaptation, t7: at re-entry; t9: 6 months after re-entry, b_1_: expectation, b_2_: experience, b_3_: expectation (quadratic), b_4_: interaction term, b_5_: experience (quadratic), a_1_ = b_1_ + b_2_ (*Do matches at high values have different outcomes than matches at low values?*), a_2_ = b_3_ + b_4_ (*Do matches at extreme values have different outcomes than matches at less extreme values?*), a_3_ = b_1_ – b_2_ (*Is one mismatch [X > Y] better or worse than the other [X < Y]?*), a_4_ = b_3_ – b_4_ + b_5_ (*Are matches better or worse than mismatches?*), explanations for a_1_ – a_4_ are verbatim quotes from Barranti et al. (2017, p. 469).

**Figure S1**


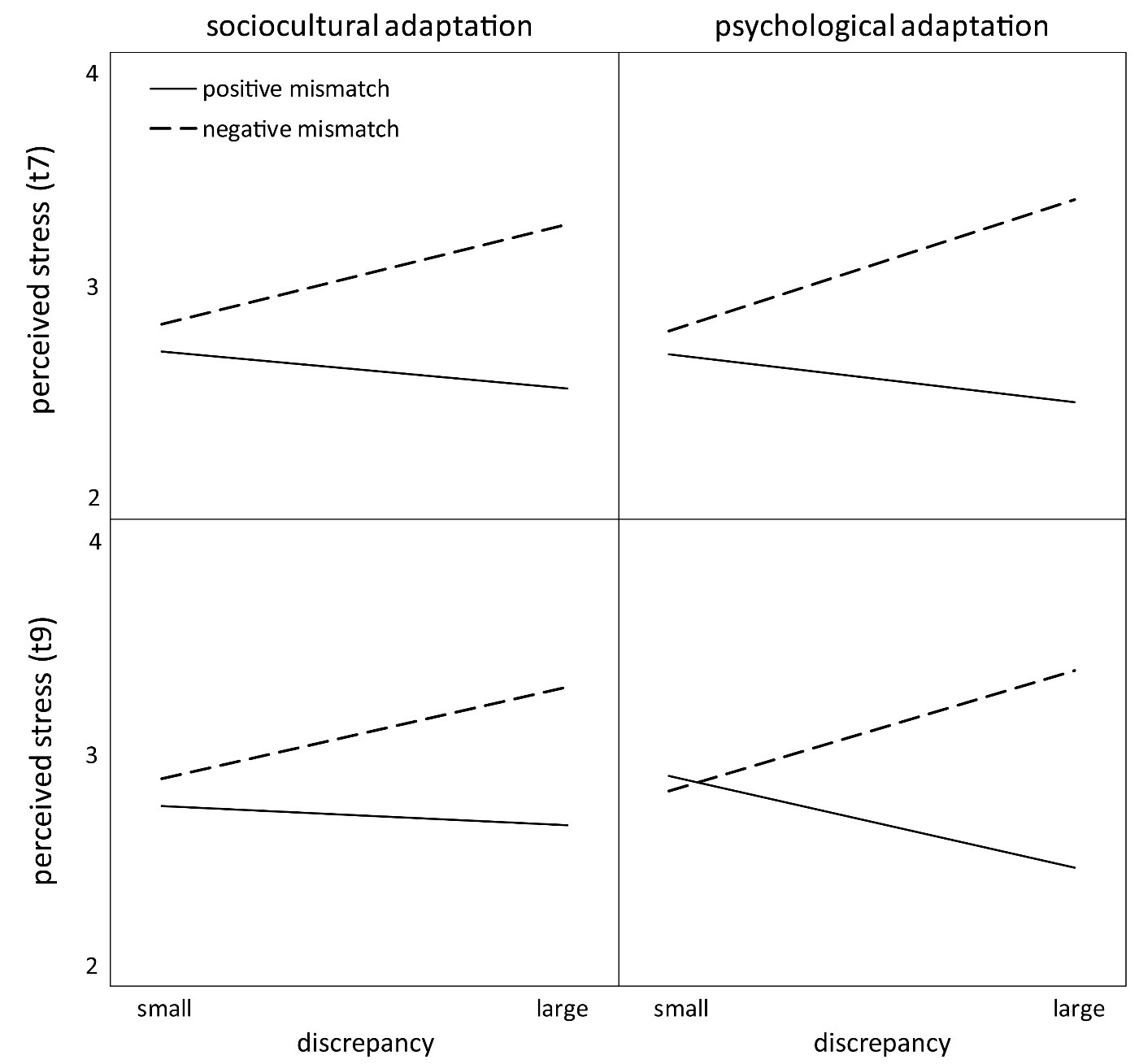


Figure S1. Simple slope analyses showing levels of perceived stress at re-entry (t7, top panels) and 6 months after re-entry (t9, bottom panels) as a function of magnitude (small to large) and direction (negative vs positive mismatch) of the discrepancy between re-entry expectations (t6) and experience of sociocultural (left panels) and psychological adaptation (right panels)

**Figure S2**


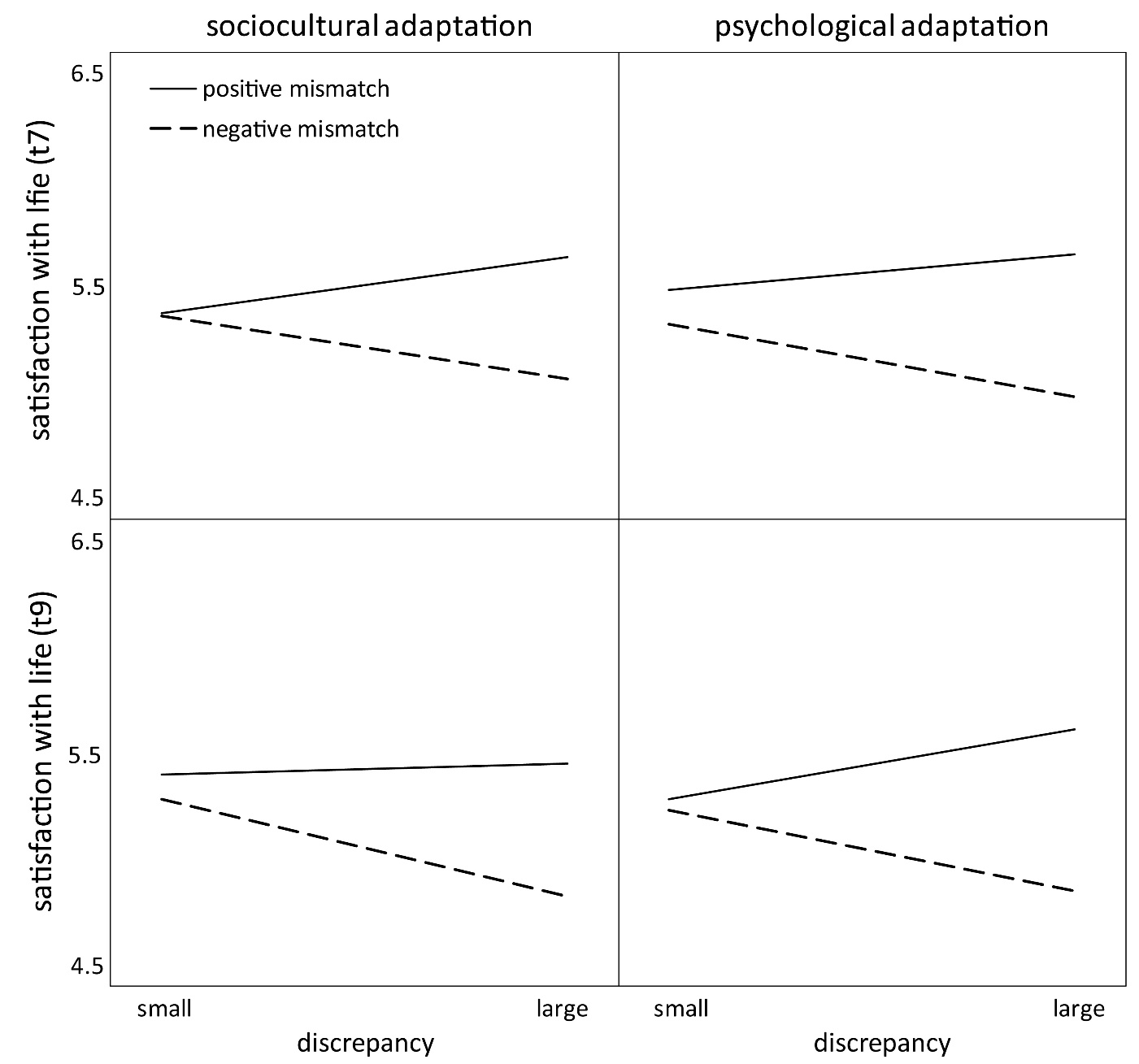


Figure S2. Simple slope analyses showing levels of satisfaction with life at re-entry (t7, top panels) and 6 months after re-entry (t9, bottom panels) as a function of magnitude (small to large) and direction (negative vs positive mismatch) of the discrepancy between re-entry expectations (t6) and experience of sociocultural (left panels) and psychological adaptation (right panels)

**Figure S3**


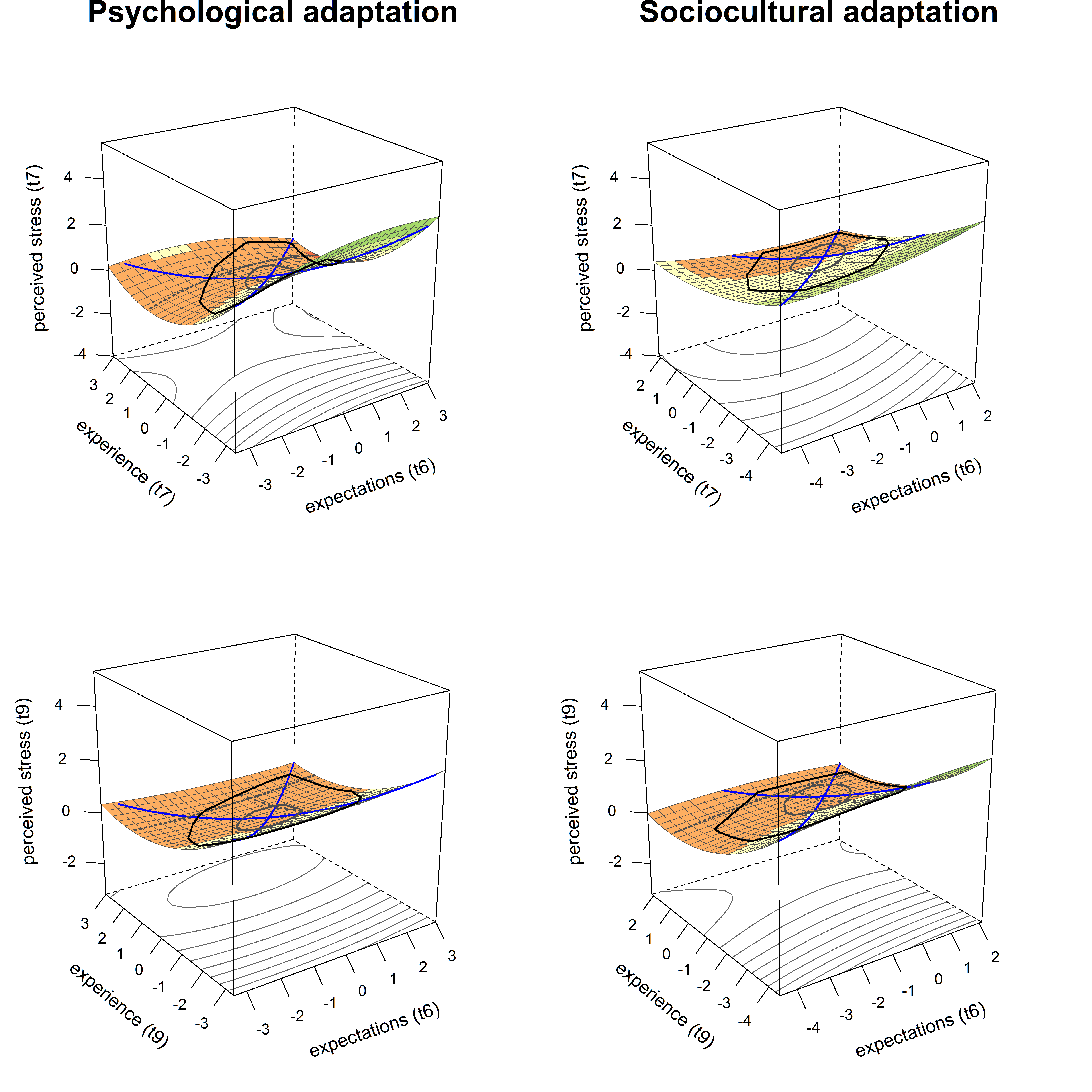


Figure S3. Three-dimensional association between expected and experienced sociocultural (left panels) or psychology adaptation (right panels) with perceived stress at re-entry (t7, top panels) and 6 months after re-entry (t9, bottom panels), controlling for perceived stress at t6.

**Figure S4**


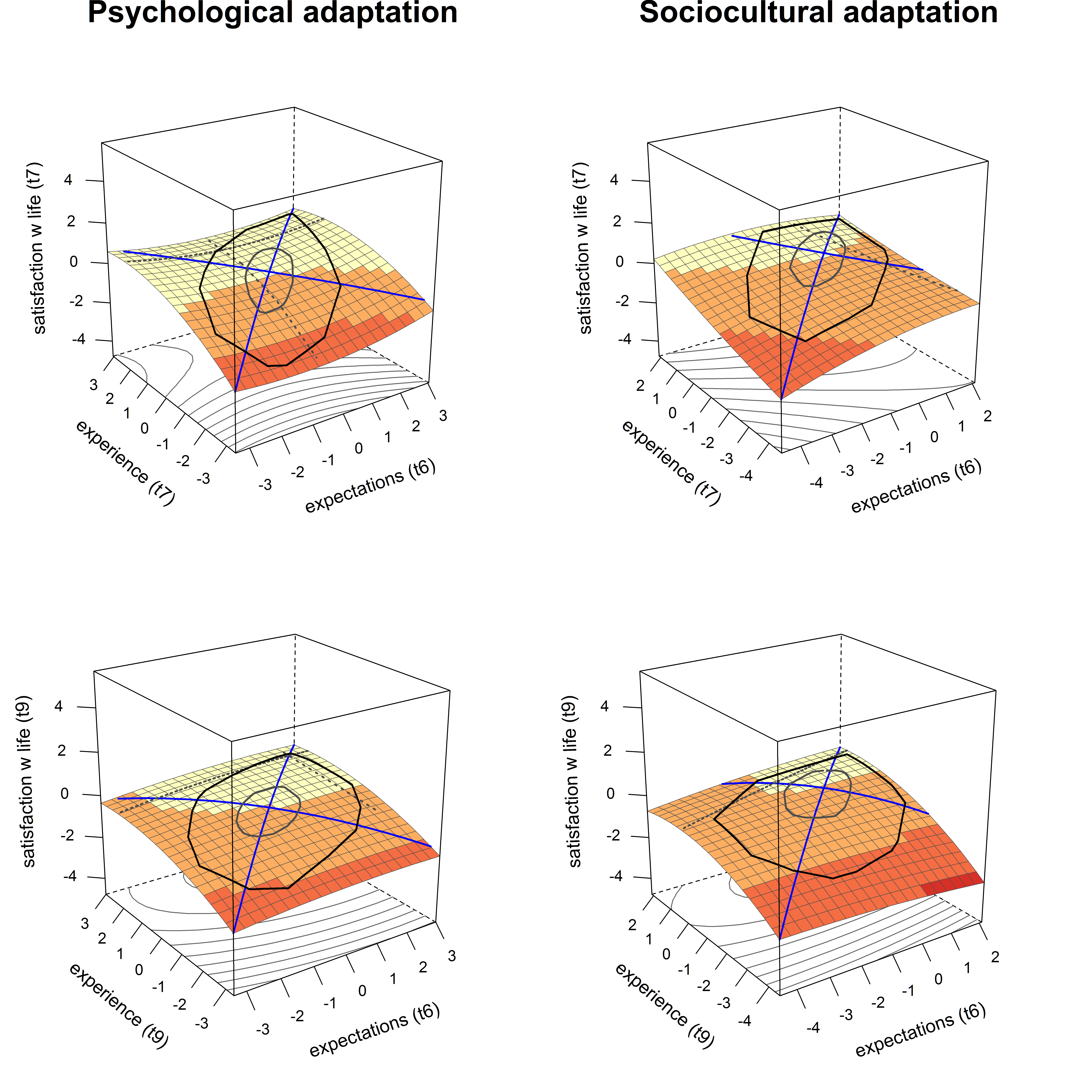


Figure S4. Three-dimensional association between expected and experienced sociocultural (left panels) or psychology adaptation (right panels) with satisfaction with life at re-entry (t7, top panels) and 6 months after re-entry (t9, bottom panels), controlling for satisfaction with life at t6.
